# Investigating the correlation between perceived stress and health anxiety with obsessive–compulsive disorder and quality of life during COVID-19 pandemic

**DOI:** 10.1186/s40359-023-01090-w

**Published:** 2023-02-28

**Authors:** Atefeh Homayuni

**Affiliations:** Student Research Committee, Hormozgan University of Medical Sciences, Bandar Abbas, Iran

**Keywords:** Anxiety, COVID-19, Obsessive–compulsive symptoms, Quality of Life, Stress

## Abstract

**Background and aims:**

The present study aimed to investigate the correlation between perceived stress and health anxiety with obsessive–compulsive symptoms and quality of life during COVID-19 pandemic.

**Methods:**

This cross-sectional study was performed in the general public in Isfahan and Bandar Abbas. 559 citizens were selected by convenience sampling. An online questionnaire was used to collect the data, which consisted of: short health anxiety inventory, perceived stress scale, world health organization quality of life questionnaire and Padua inventory. Data analysis was performed using SPSS-24 and Amos-21.

**Results:**

There were significant positive correlations between health anxiety and perceived stress (r = 0/338), obsessive–compulsive symptoms and perceived stress (r = 0/16), obsessive–compulsive symptoms and health anxiety (r = 0/344). Also there were significant negative correlations between obsessive–compulsive symptoms and quality of life (r = − 0/21), health anxiety and quality of life (r = − 0/366), perceived stress and quality of life (r = − 0/715).

**Conclusion:**

health anxiety and perceived stress during COVID-19 affect the obsessive–compulsive symptoms and quality of life. Therefore, it is recommended to pay attention to these psychological disorders during this global crisis and take actions to prevent and treat them.

## Introduction

Coronavirus (COVID-19) was first emerged in Wuhan province of China. It became a pandemic in just two months according to the World Health Organization (WHO) [1]. Based on WHO reports, up to January 31, 2023, 753,479,439 cases, including 6,812,798 deaths have been confirmed in the world [2]. During this epidemic, global health systems experience critical challenges in preventing infectious, identifying and managing COVID-19 cases, and ensuring effective strategies to protect public health [3, 4].

Uncertainty of the upcoming situation and low predictability of COVID-19 threaten individual's physical and mental health. Li et al. [5] in a study conducted on active Weibo (a leading Chinese Online Social Networks with more than 462 million active daily users in 2019) users showed that during COVID-19, the negative emotions (e.g., anxiety, depression and indignation) and sensitivity to social risks increased, while positive emotions (e.g., happiness and life satisfaction) decreased. People's concern about their health, family, death and religion increased, but it decreased in leisure and friends. The results of a review study conducted by Hossain et al. [6] showed that people affected by COVID-19 may have a high burden of mental health problems, including depression, anxiety disorders, stress, panic attack, irrational anger, impulsivity, somatization disorder, sleep disorders, emotional disturbance, posttraumatic stress symptoms, and suicidal behavior.

Studies showed that stresses and worries caused by COVID-19 pandemic may reduce the quality of life [7]. The majority of participants in Horesh et al.'s [8] study reported relatively high levels of perceived stress and corona-related worries. Female gender, younger age, corona-related loneliness and pre-existing chronic illness were related to higher levels of psychological distress and lower levels of quality of life. The results of a study conducted by Peters et al. [9] showed that persons with children under 12 years of age showed significantly higher stress levels than others and their health-related quality of life was comparable.

The stress caused by the COVID-19 virus also has caused many problems for people who are vulnerable to obsessive–compulsive disorder (OCD), to the extent that cleaning and washing is carried out in an extreme manner and disrupts the normal course of their lives. Since COVID-19 was declared as a pandemic by WHO with strict emphasis on washing and disinfection, reporting of patients with OCD and their difficulties were increased [10]. Recent works [11, 12] have shown that individuals from general public experience difficulties to re-adjust to normal ways of living even after lockdown is released, and those with obsessive–compulsive (OC) symptoms and traits are disproportionally affected. Participants in Hassoulas et al.'s [13] study reported experiencing overwhelming fear and anxiety during the pandemic, leading to worsening in contamination-elated compulsive behaviors.

Health anxiety occurs when perceived feelings or physical changes in a person are interpreted as symptoms of an illness, which may include physical symptoms such as fever, cough, and muscle aches associated with an infectious disease [14, 15]. In the conditions of the spread of the viral diseases (such as Ebola, H1N1 influenza and COVID-19), people with high health anxiety may misinterpret benign muscle aches or even mild common coughs as signs that they are infected. This will, in turn, increase their anxiety [16, 17]. Roy et al. [18] showed that people experienced more anxiety and health anxiety during COVID-19.

Because of the disease's resiliency, many individuals with contamination fears will experience increased anxiety and distress over acquiring this disease. Contamination-fearful individuals cope with their fears by taking sanitary measures such as washing hands, using bleach, and wearing masks, compulsions that can actually worsen the problem [19]. Jelinek et al. [20] found that OCD symptoms increased during COVID-19. This increase was stronger in washers compared to non-washers. Dysfunctional hygiene-related beliefs were significantly higher in washers than non-washers and were associated with greater symptom progression.

Delay in starting vaccination, people’s negligence in observing health protocols and their resistance to vaccination, the existence of a lot of tourism attractions in Isfahan and the industrial nature of this city, high humidity in BandarAbbas which makes it difficult for people to use face masks outdoors and the cultural background of people, who are known to hospitable, contributes to the high prevalence of covid-19 and further psychological problems in these cities. Consequently, in order to creating effective interventions to improving people's mental health during COVID-19, identifying the relevant and predictive psychological factors is essential. In this regard, the present study was conducted to evaluate the correlations between perceived stress and health anxiety with obsessive–compulsive symptoms and quality of life during COVID-19 pandemic. The following hypotheses are proposed:

1. Perceived stress can predict quality of life during COVID-19 pandemic.
2. Health anxiety can predict quality of life during COVID-19 pandemic.
3. Perceived stress can predict OC symptoms during COVID-19 pandemic.
4. Health anxiety can predict OC symptoms during COVID-19 pandemic.

## Materials and methods

The population of this descriptive-correlational study consisted of residents over 16 years old in Isfahan and Bandar Abbas. The sample size was calculated based on the following formula:

$$n = \left[ {(z_{1 - \alpha /2} + z_{1 - \beta } )/C} \right]^{2} + 3\,\,\,,\,\,\,C = 0.5*\ln [(1 + r)/(1 - r)]$$

Assuming a 5% error, 90% test power and a correlation coefficient of -0.18 in previous studies [21], the sample size was estimated at 320 people based on the formula. Considering the design effect of 1.2 for cluster sampling, the final sample size was estimated at 384.

The inclusion criteria for the sample selection included: literacy, access to internet to answer questions, not suffering from psychological disorders such as obsessive–compulsive disorder, the minimum age of 16 years and living in Isfahan or Bandar Abbas. Exclusion criteria included people with psychological disorders such as obsessive–compulsive disorder and unwillingness to participate in the study.

Regarding to the existing limitations due to the outbreak of COVID-19 and the impossibility of distributing questionnaires in paper form, data were collected using a questionnaire designed on the Pors Line platform, an online survey platform in Iran and was provided to the target group through social media (such as WhatsApp, Telegram), emails, channels and new agencies and public relations of University of medical sciences. On the first page of the questionnaire, after explaining the objectives of the study, the participants were asked to sign a consent form. We assured them to keep their information confidential by the research team and use the data obtained from the research only for statistical analysis in line with research objectives.

### Instruments

The following demographic questionnaire and self-administered tools were used to collect data. The assessed demographic characteristics were gender, marital status, education level, age, employment status and chronic diseases.

#### Short health anxiety inventory (SHAI)

This inventory is an 18-item self-report scale. Each of the 18 items consists of four statements, in which individuals select the one that best reflects their feelings during the last six months. The responses are scored on a 4-point Likert scale (0–3 scores). This questionnaire enables estimating the magnitude of health anxiety in two components: the probability of getting the disease (14 questions) and the negative consequences of getting the disease (4 questions). Overall scores ranged from 0 to 54, whereby the higher scores indicated the higher health anxiety [22]. The reliability coefficient through test–retest method with a one-week interval has been obtained at 0.90 [23]. In the present study, Cronbach's alpha coefficient for this questionnaire was 0.855.

#### Perceived Stress Scale (PSS)

The Perceived Stress Scale is a 14-item self-report tool developed by Cohen et al. (1983) to measure general perceived stress, thoughts and feelings about stressful events, control, overcoming, coping with stress, and experienced stress over the past month. On this scale, individuals are asked to indicate their feelings over the past 4 weeks on a 5-point Likert scale from 0 (never) to 4 (always). This scale measures two subscales: (a) perceived helplessness, which includes items 1, 2, 3, 8, 11, 12, and 14. (b) perceived self-efficacy, which includes items 4, 5, 6, 7, 9, 10, and 13, and these items are scored inversely. The lowest score is zero and the highest score is 56. The higher scores indicated the higher perceived stress. In a study conducted by Cohen et al. (1983), the internal consistency coefficients for each of the subscales and the general PSS were between 0.84 to 0.86 [24]. In the present study, Cronbach's alpha coefficient for this questionnaire was 0.887.

#### World Health Organization Quality of Life Questionnaire (WHOQOL-BREF)

This questionnaire is a 26-item instrument consisting of four domains: physical health (7 items), psychological health (6 items), social relationships (3 items) and environmental health (8 items). The first two questions assess the general health and quality of life. Each question is scored on a 5-point Likert scale (1 = strongly disagree to 5 = strongly agree). The score of each aspect is calculated separately from the total score of its questions, so that score 4 indicates the worst and score 20 indicates the best situation in that aspect [25]. Yousefy et al. [26]) reported Cronbach's alpha coefficient for physical health, psychological health, social relationships and environmental health were 0.81, 0.72, 0.78, and 0.76, respectively. In the present study, Cronbach's alpha coefficient for this questionnaire was 0.932.

#### Padua inventory

To measure washing compulsions and contamination obsessions, Padua Questionnaire (Modified by Washington State University) was used [27]. Subjects' responses to each item are scored on a 5-point Likert scale (0 = not at all, 1 = low, 2 = somewhat, 3 = high, and 4 = very high). High scores indicated a high level of obsessive–compulsive symptoms in the subject. Van Open [28] obtained obsessive–compulsive symptoms coefficients at 0.94 for Padua questionnaire and above 0.80 for its subscales. In the present study, Cronbach's alpha coefficient for this questionnaire was 0.875.

### Statistical analysis

Data were analyzed in SPSS-24 and Amos-21. To test the hypotheses and discover the correlations between the variables, Pearson correlation analysis, path analysis and structural equation modeling (SEM) were used. The level of significance was considered to be 95% (*p* < 0.05).

### Ethics statement

Ethical approval was received for this study from the Ethics Committee of the Hormozgan University of Medical Sciences (IR.HUMS.REC.1399.439). Written informed consent was obtained from individuals who participated in this study. For participants between 16 and 18 years of age, the research ethics committee (Ethics Committee of the Hormozgan University of Medical Sciences) waived the requirement for parental consent.

## Results

A total of 559 subjects with the mean age of 37.34 ± 11.19 years participated in this study. 21.5% of the participants were male and 78.5% were female. More than half of the participants were married (71.6%) and the rest were single (26.8%), divorced (1.1%) or widow (0.5%). The majority of them had bachelor's degree (42.9%) and the rest have high school (6.5%), diploma (17.9%), associate degree (7.7%) and master's degree and higher (25%). In relation to job situation, 40.9% of the participants were employed and the rest were student (3.6%), university student (7%), unemployed (4.5%), housewife (32.4%) and self-employed (11.6%). Only 14.5% of the participants were members of the medical, health and treatment staff and 83.4% of them had no history of chronic diseases.

The correlation matrix of the variables is presented in Table 1.

**Table 1:** correlation coefficients of study variables

variables	Mean (SD)	1	2	3	4	5	6	7	8	9	10
Perceived helplessness	13.92 ± 5.41		0.541^**^	0.897^**^	0.327^**^	0.303^**^	0.355^**^	0.147^**^	0.19^**^	0.184^**^	− 0.597^**^
Perceived self-efficacy	14.43 ± 4.65			− 0.857^**^	− 0.186^**^	− 0.279^**^	− 0.231^**^	− 0.071	− 0.094^*^	− 0.9^*^	0.665^**^
Perceived stress	27.49 ± 8.83				0.298^**^	0.332^**^	0.338^**^	0.127^**^	0.166^**^	0.16^**^	− 0.715^**^
Risk of disease	12.32 ± 6.42					0.508^**^	0.973^**^	0.328^**^	0.281^**^	0.339^**^	− 0.302^**^
Negative effect of disease	1.83 ± 2.06						0.693^**^	0.221^**^	0.18^**^	0.224^**^	− 0.424^**^
Health anxiety	14.15 ± 7.67							0.334^**^	0.283^**^	0.344^**^	− 0.366^**^
Contamination obsessions	10.69 ± 5.54								0.634^**^	0.923^**^	− 0.187^**^
Washing compulsions	9.01 ± 4.53									0.883^**^	− 0.193^**^
Obsessive–compulsive symptoms	19.7 ± 9.12										/0.21^**^
Quality of life	87.03 ± 15.81										

^**^Correlation is significant at the 0.01 level

^*^Correlation is significant at the 0.05 level

The fit indices of the model are presented in Table 2.

**Table 2:** Model fit indices-Goodness of fit

RMSEA	AGFI	GFI	NFI	NNFI	CFI	CMIN/DF
0.075	0.921	0.958	0.954	0.945	0.964	4.098

According to the results of Table 2, the fit indices to evaluate the totality of the final model indicated that in general the model has appropriate fitness.

**Fig. 1:**
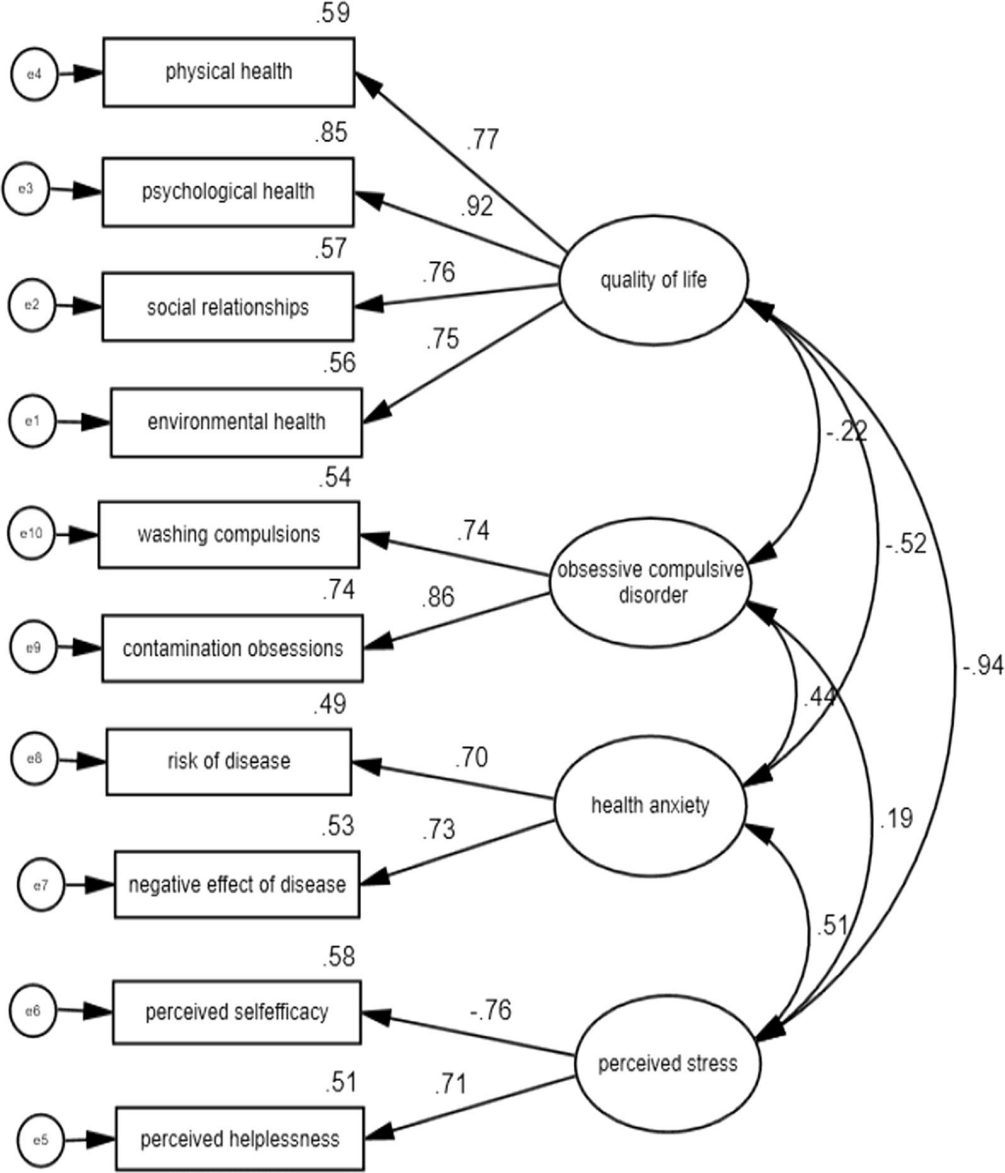
The structural equation modeling (SEM) of psychological problems during coronavirus

The results of structural equation modeling are presented at Fig. 1.

As seen in Table 3, the subscales of environmental health with a standardized estimate of 0.747, social relationships with a standardized estimate of 0.757, psychological health with a standardized estimate of 0.923, and physical health with a standardized estimate of 0.767 explain the quality of life. Perceived helplessness with a standardized estimate of 0.713 and perceived self-efficacy with a standardized estimate of − 0.759 explain perceived stress. Negative effect of disease with a standardized estimate of 0.726 and risk of disease with a standardized estimate of 0.7 explain health anxiety, and contamination obsessions with standardized estimation of 0.861 and washing compulsions with a standardized estimate of 0.736 explain obsessive–compulsive symptoms.

**Table 3:** Regression weight & standardized regression weight

			Estimate	S.E	C.R	*P*	Standardized Estimate
Environmental health	←	Quality of life	1.000				0.747
Social Relationships	←	Quality of life	0.492	0.027	18.061	< 0.001	0.757
Psychological health	←	Quality of life	1.081	0.049	22.118	< 0.001	0.923
Physical health	←	Quality of life	0.946	0.052	18.324	< 0.001	0.767
Perceived helplessness	←	Perceived stress	1.000				0.713
Perceived self-efficacy	←	Perceived stress	− 0.914	0.056	− 16.265	< 0.001	− 0.759
Negative effect of disease	←	Health anxiety	1.000				0.726
Risk of disease	←	Health anxiety	3.010	0.301	10.014	< 0.001	0.700
Contamination obsessions	←	obsessive compulsive symptoms	1.000				0.861
Washing compulsions	←	obsessive compulsive symptoms	0.700	0.088	7.96	< 0.001	0.736

As seen in Table 4, there is a significant correlation between all variables in pairs. There is a negative correlation between obsessive–compulsive symptoms and quality of life (r = − 0.222). There is a negative correlation between health anxiety and quality of life (r = − 0.524). There is a positive correlation between health anxiety and perceived stress (r = 0.513). There is a negative correlation between perceived stress and quality of life (r = − 0.941). There is a positive correlation between obsessive–compulsive symptoms and perceived stress (r = 0.19) and finally, there is a positive correlation between obsessive–compulsive symptoms and health anxiety (r = 0.439).

**Table 4:** Covariances and correlations

			Estimate	S.E	C.R	*P*	Correlations
Quality of life	↔	Obsessive compulsive symptoms	− 4.121	0.961	− 4.289	< 0.001	− 0.222
Quality of life	↔	Health anxiety	− 3.038	0.384	− 7.903	< 0.001	− 0.524
Perceived stress	↔	Health anxiety	2.953	0.412	7.175	< 0.001	0.513
Quality of life	↔	Perceived stress	− 14.097	1.228	− 11.480	< 0.001	− 0.941
Perceived stress	↔	Obsessive compulsive symptoms	3.500	1.060	3.303	< 0.001	0.190
Health anxiety	↔	Obsessive compulsive symptoms	3.126	0.468	6.678	< 0.001	0.439

## Discussion

The present study investigated the correlation between perceived stress and health anxiety with obsessive–compulsive symptoms and quality of life. The results showed that there is a negative correlation between health anxiety and quality of life. This finding is consistent with findings from other studies conducted by Abdelghani et al. [29], Trougakos et al. [30] and Korkmaz et al. [31]. Abdelghani et al.[29] in a cross-sectional study conducted on health care staff in hospitals in Egypt showed that health anxiety to COVID-19 virus was inversely correlated with all domains of quality of life. Trougakos et al. [30] showed that COVID-19 health anxiety increase emotion suppression and lack of psychological need fulfillment and as a result impair health, home and job outcomes. The results of the study conducted by Heidari Shams et al. [32] on outpatients of Shahid Beheshti Specialized Polyclinic in Iran showed that there is a significant negative correlation between quality of life and health anxiety. High levels of health anxiety during the COVID-19 affect the people's rational decisions and the occurrence of maladaptive behaviors, which in turn leads to distress, social disability, occupational dysfunction, and frequent visits to health centers [33] and consequently reduced quality of life.

The results showed that there is a positive correlation between health anxiety and obsessive–compulsive symptoms. This finding is consistent with previous studies conducted by Hong et al. [34], Wheaton et al. [35], Hassoulas et al. [13] and Sica et al. [36]. Hong et al. [34] found that there is a positive correlation between health anxiety with obsessive–compulsive symptoms during the COVID-19 pandemic. Sica et al. [36] revealed that in addition to health anxiety, people with washing and contamination obsession may also be susceptible and vulnerable to COVID-19 fears. These individuals may be at risk of exacerbation of obsessive–compulsive symptoms during COVID -19. Previous studies have shown that health anxiety can significantly encourage OC symptoms [37]. According to the cognitive model of health anxiety, during the COVID-19 pandemic, individuals with health anxiety continued to adopt a series of maladaptive and repetitive behaviors which were associated with obsessive–compulsive symptoms (including over-washing, over-checking, obsessing, and metal neutralizing) [34]. In other words, people with high health anxiety engaged in a variety of maladaptive safety behaviors, including obsessive hand washing, social isolation, and shopping with panic. In general, obsessive thoughts cause anxiety, while obsessive action related to it, is performed to reduce anxiety [38]. Moreover, Hypochondriasis, which is a more severe form of health anxiety, has been recently reclassified under Obsessive–Compulsive and Related Disorders in ICD-11 and a recent meta-analysis has shown that the same treatments which are effective for OCD (CBT and SSRI), are also effective for Hypochondriasis [39].

The results revealed a negative correlation between perceived stress and quality of life. This result is in line with the results of studies conducted by Çelmeçe and Menekay [40], Tejoyuwono et al. [41], and Qi et al. [42]. The results of the study conducted by Qi et al. [42] on the Chinese adult population during the COVID-19 pandemic showed that perceived stress was negatively correlated with physical component summary and mental component summary for health related quality of life. Alhawtmeh et al. [43] in a cross-sectional study conducted on Jordanian registered nurses during the COVID-19 pandemic, showed that perceived stress was negatively correlated with quality of life. Khodami et al. [44] found that quality of life is significantly decreased over time, meanwhile perceived stress level is raised significantly. Younger people and individuals who had a worsening quality of life response tended to show more stress. During the quarantine, people's routines are disrupted and as a result, they are less able to predict and plan for their future. People feel that the amount of control they have over their lives is reduced and this situation causes them insecure feeling. This insecurity causes stress. On the other hand, fear of illness, fear of death, fear of financial problems and loss of job, uncertainty about the status of disease, uncertainty about time of disease control and etc. cause stress and anxiety, which may affect the people's quality of life during this pandemic.

The results showed that there is a positive correlation between perceived stress and health anxiety. The results of a study conducted by Garboczy et al. [45] among university students amid to COVID-19 pandemic showed that there was a significant positive relationship between perceived stress and health anxiety. In such situations, there is a tendency to use coping mechanisms other than appropriate mechanisms, which in turn can lead to an increase in perceived stress levels [45]. Another study conducted in the UK during the first peak of COVID-19 pandemic showed a strong positive association between health anxiety and perceived stress. Stress and health anxiety scores reached the peak level in the first weeks of the imposed quarantine. With extending the quarantine, participants reported lower scores on stress, health anxiety, and community cohesion [46]. In situations such as epidemics, where there are many psychological challenges (such as worrying about essentials such as food availability and inability to plan long-term life), people with high stress experience higher anxiety [47].

The results also revealed a positive correlation between perceived stress and obsessive- compulsive symptoms. Studies have indicated that the prevalence of perceived stress, anxiety, depression, and obsessive–compulsive symptoms increased significantly during the Covid-19 epidemic [48]. The results of a study conducted by Abba-Aji et al. [49] in a Canadian province showed that the prevalence of OCD symptoms increased during the COVID-19 pandemic. There were significant correlations between obsessions about dirt, germs and viruses, and between those who engaged in compulsive hand washing and the likelihood that respondents had moderate/high stress. Increased general stress (for example, job loss and family disease) and changes in normal life routines are among the causes of severe obsessive–compulsive symptoms [50], and it is true about COVID-19 and the quarantine caused by it.

The results showed a negative correlation between obsessive–compulsive symptoms and quality of life. This finding is consistent with previous studies conducted by Cunning and Hodes [51] and Guzick et al. [52]. Cunning and Hodes [51] found that OC symptoms were exacberated during the COVID-19 pandemic. Guzick et al. [52] found that OC symptoms worsened during the early stages of the COVID-19 pandemic. Many patients and individuals in the general population experienced new obsessive–compulsive-like symptoms centered on COPVID-19.

The limitations of this study included the following cases: First, the data were collected online and only literate people with internet access were able to participate in the study and the findings may be prone to selection bias. Second, psychological health was reported by the participants and acceptable assessment tools were not used. Another limitation is that we did not include in the analyses other psychological variables such as history of mental health disorder; therefore, in order to corroborate our results, larger studies including those psychological variables, are needed. This study is recommended in other populations and with other psychological variables.

## Conclusion

The current study showed that COVID-19 pandemic affected people's mental health negatively, which in turn decreased their quality of life. Considering the importance of psychological health of people during this pandemic, identifying psychological disorders and investigating its possible impact on the quality of life will help policymakers and health professionals to maintain and improve people's mental health by providing appropriate psychological solutions and strategies.

## Data Availability

The datasets generated and analyzed during the current study are not publicly available due to confidentiality and privacy related issues but are available from the corresponding author on reasonable request.

## Abbreviations

OC: Obsessive–compulsive
OCD: Obsessive–compulsive disorder
PSS: Perceived Stress Scale
SEM: Structural equation modeling
SHAI: Short health anxiety inventory
WHO: World Health Organization
WHOQOL: World Health Organization Quality of Life
